# High resolution analysis of the human transcriptome: detection of extensive alternative splicing independent of transcriptional activity

**DOI:** 10.1186/1471-2156-10-63

**Published:** 2009-10-05

**Authors:** Weiyin Zhou, Margaret A Calciano, Heather Jordan, Michael Brenner, Seth Johnson, Darong Wu, Lin Lei, Diego Pallares, Pascale Beurdeley, Fabien Rouet, Pritmohinder S Gill, Laurent Bracco, Cyril Soucaille, Richard Einstein

**Affiliations:** 1 ExonHit Therapeutics Inc, 217 Perry Parkway, Bldg 5, Gaithersburg, MD, 20877 USA; 2 ExonHit Therapeutics SA, 65, blvd Massena F-75013 Paris, France

## Abstract

**Background:**

Commercially available microarrays have been used in many settings to generate expression profiles for a variety of applications, including target selection for disease detection, classification, profiling for pharmacogenomic response to therapeutics, and potential disease staging. However, many commercially available microarray platforms fail to capture transcript diversity produced by alternative splicing, a major mechanism for driving proteomic diversity through transcript heterogeneity.

**Results:**

The human Genome-Wide SpliceArray™ (GWSA), a novel microarray platform, utilizes an existing probe design concept to monitor such transcript diversity on a genome scale. The human GWSA allows the detection of alternatively spliced events within the human genome through the use of exon body and exon junction probes to provide a direct measure of each transcript, through simple calculations derived from expression data. This report focuses on the performance and validation of the array when measured against standards recently published by the Microarray Quality Control (MAQC) Project. The array was shown to be highly quantitative, and displayed greater than 85% correlation with the HG-U133 Plus 2.0 array at the gene level while providing more extensive coverage of each gene. Almost 60% of splice events among genes demonstrating differential expression of greater than 3 fold also contained extensive splicing alterations. Importantly, almost 10% of splice events within the gene set displaying constant overall expression values had evidence of transcript diversity. Two examples illustrate the types of events identified: LIM domain 7 showed no differential expression at the gene level, but demonstrated deregulation of an exon skip event, while erythrocyte membrane protein band 4.1 -like 3 was differentially expressed and also displayed deregulation of a skipped exon isoform.

**Conclusion:**

Significant changes were detected independent of transcriptional activity, indicating that the controls for transcript generation and transcription are distinct, and require novel tools in order to detect changes in specific transcript quantity. Our results demonstrate that the SpliceArray™ design will provide researchers with a robust platform to detect and quantify specific changes not only in overall gene expression, but also at the individual transcript level.

## Background

A large portion of the diversity within the transcriptome is generated by alternative splicing, which in some cases, can produce thousands of transcripts from a single gene or locus [1,2]. This has important implications in biology and pathophysiology where extensive alterations in transcripts resulting from alternative splicing produce structurally different products and impacts the function of genes in biology, disease [3-6], as well as processes such as evolution [7,8]. The fine granularity of the transcriptome has not been determined with clarity, and new commercial tools are required in order to begin to identify with certainty the diverse content of the transcriptome. Traditional microarray designs and analytical methods are not robust enough to detect this transcript diversity. SpliceArray™ microarrays were developed to experimentally define the composite of transcripts that are present within biological samples and have the ability to detect subtle differential changes in gene expression for different alternatively spliced isoforms. The performance parameters of the human Genome-Wide SpliceArray™ (GWSA) which monitors over 280,000 potential splicing events (known and predicted) were assessed here, guided by the recent publications from the MicroArray Quality Control (MAQC) consortium.

The MAQC project [9] is a FDA sponsored consortium founded to address concerns of microarray reproducibility of expression profiling experiments. The purpose was to generate quality control tools for the microarray community in order to avoid procedural failures and to develop guidelines for microarray data analysis by providing the public with large reference datasets along with readily accessible reference RNA samples. The study found, that overall, the platforms perform similarly [9] and were validated with alternative quantitative gene expression platforms [10]. However, all the platforms tested contain a similar bias where probes were designed to monitor the overall level of the gene, and do not give any expression information toward the isoform diversity produced from each gene through alternative splicing. Here we present a novel microarray platform and analysis for the efficient detection of isoform diversity on a genome wide scale in human. Our analysis of this new microarray design is in accordance with the approach outlined by the MAQC consortium and demonstrates that the SpliceArray™ products are highly reproducible, quantify transcripts, and are sensitive in detecting subtle changes in transcript ratios.

## Results

Alternative splicing events were identified through a comparison of sequence data from the NCBI GenBank database. A total of 20,649 human genes were selected and analyzed for representation on the human Genome-Wide SpliceArray™ (GWSA). From this group, 19,066 genes or 92.3% were found to contain evidence of potential alternative splicing within the selected sequence collection. Table 1 lists the complete distribution of the different event types identified and included on the GWSA. For each splice event a series of probe sets (F, T, B, C, D, and E) consisting of up to three probes per probe set were generated to detect different transcripts or isoforms (Additional file 1; [11,12]). In addition to the F, T, B, C, D and E probe sets that monitor the ~138,000 cDNA evidenced splice events and ~142,000 predicted exon skip events, a series of structural probe sets are included on the human GWSA that monitor ~176,000 exon-intron boundaries for novel alterations (Additional file 1; Table 1).

**Table 1:** Distribution of splice events from an analysis of the human genome.

**Event Type**	**Number of events**
Total putative alternative splicing events with cDNA supporting evidence:	**138,636**
▪ novel exon	46,352
▪ novel exons	9,413
▪ exon skipped	31,163
▪ exons skipped	11,905
▪ alternative splice donor (ASD)	10,281
▪ alternative splice acceptor (ASA)	12,606
▪ intron retention	5,999
▪ novel intron	10,917
Predictions of single exon skips with no supporting cDNA evidence	**142,697**

Intron/exon boundary regions with structural probes for prediction of ASA, ASD and intron retentions	176,000

Results from the human genome analysis of splicing events from 20,649 genes are tabulated according to the type of splice event. All events included here are monitored on the human GWSA. The alternative splicing results for the complete transcriptome data set are available online at .

To assess performance of the human GWSA, samples were selected in accordance with the MAQC project, which included human universal reference RNA (Sample A), human brain RNA (Sample B) and two titration samples (C and D; see methods; refer to fig. 1 in [13]). As with the MAQC project [13], we assumed a linear response for each probe across the four titration samples (A-D). The frequency distribution of the expression values for all 12 arrays (samples A-D run in triplicate) showed a normal distribution of signal as expected for the platform (Additional file 2-A). The reproducibility of the arrays was found to be highly concordant and produced low coefficients of variation (CV%) for each sample analyzed. The mean CV% were found to be slightly higher than the median values (5.5 versus 4.23, respectively), indicating a slight bias towards higher values. On a gross level, principal component analysis (Additional file 2-B) showed close grouping of the replicates.

**Figure 1:**
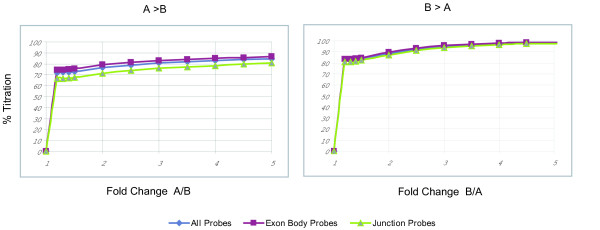
**Titration analysis of probe set response**. Probe sets were selected for analysis based on the expression results detecting different transcripts. Probe sets were binned based on the fold change between samples A (universal reference) and B (total brain), and assessed for correct titration of expression values for samples A, B, C, and D. Probe sets were filtered statistically and those with p values < 0.001 were analyzed. Probe types were separated according to exon body and junction probes. The left graph indicates probe sets which were more highly expressed in sample A than sample B (A > B), and the right graph displays probe sets which were more highly expressed in sample B versus sample A (B > A).

The titration samples were analyzed at the probe set, gene, and event level to define the quantitative nature of the expression data generated by the array. After filtering low-expressing probe sets, 527,574 probe sets were identified in which expression was higher in the universal sample (A) than in the brain sample (B) (A>B) and 472,085 probe sets where B>A. Based on the fold change between samples A and B, probe sets were assessed for a correct titration response of expression values such that where the probe set showed a fold change of A > B, that it also demonstrated that sample A > sample C > sample D > sample B. Where probe sets were found to be B > A, calculations were made to identify probe sets were B > D > C > A. Exon body probe sets demonstrated the ability to titrate slightly more efficiently than junction probe sets for both groups and were more efficient in detecting the proper response (Figure 1).

**Figure 2:**
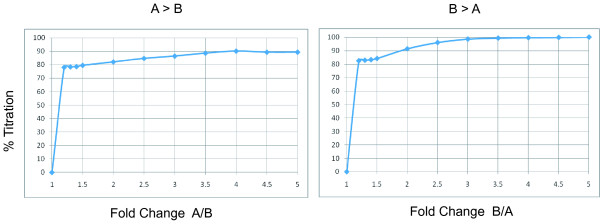
**Titration response based on gene level analysis**. To calculate the gene level expression values, the median expression value was calculated from probe sets common to both isoforms (the F and T probes). The fold change for each gene was determined between samples A and B, binned and assessed for correct titration of expression values among the four samples. Genes were filtered statistically and those with p values < 0.001 were analyzed. The left graph indicates genes which were more highly expressed in sample A than sample B (A > B), and the right graph displays genes which were more highly expressed in sample B versus sample A (B > A).

**Figure 3:**
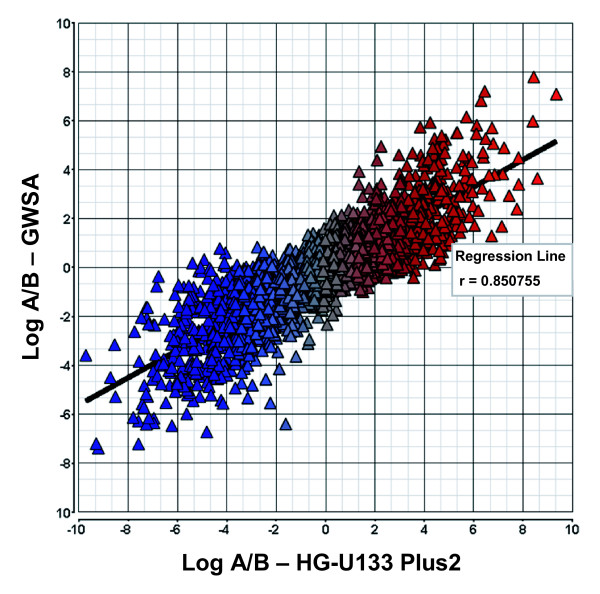
**Gene level comparison of human GWSA with the HG-U133 Plus 2.0 array**. Gene annotations from the human GWSA were mapped against the U133 Plus 2.0 array and after removing the low expressed genes, 7147 genes remained for comparative analysis. Fold changes for each gene were calculated between samples A and B for each platform and plotted against each other. Correlation coefficients (found in table 1) were calculated based on fold change gene sets. A direct comparison of the intensity values was prevented due to the significant differences between the platforms (5 micron versus 11 micron feature size, different labeling technology, and probe sequences).

The probe set configuration was used to generate total gene level expression values which were calculated from the array data using the probes F and T which are common to most isoforms of the gene and as such represent all common features for all transcripts at a locus. On average, 87% of genes that were significantly differentially expressed between samples A and B (p < 0.001) and had greater than a 2.0 fold change demonstrated correct titration of all four samples (Figure 2), indicating the array quantifies gene expression at the gene level in an accurate manner. In addition, we compared gene expression levels from data generated on the GWSA to a more conventional, 3' biased microarray, the Affymetrix HG-U133 Plus 2.0 GeneChip. The Affymetrix HG-U133 Plus 2.0 GeneChip data set taken from the MAQC Project included only 12,091 genes [13] which were mapped back to our data on the human GWSA. Comparison of the annotations from each array provided 11,089 out of a possible 12,091 genes in common which was reduced to 7,147 genes after removing genes exhibiting low expression. As expression values generated on different platforms cannot be directly compared due to different platform parameters, labeling methods, probe sequences, etc., we compared the relative expression difference between samples A and B. The log ratio of sample A versus sample B for all genes was highly correlated between the two platforms with a linear correlation of 0.85 (Figure 3, Table 2). When considering genes that showed a 2 fold change between samples, the coefficient rose to 0.90, and continued to rise as the fold change increased (Table 2), indicating that the human GWSA provides highly concordant quantification of gene expression when compared to the Affymetrix HG-U133 Plus 2.0 GeneChip.

**Table 2:** Correlation of gene level analysis between the human GWSA and the Affymetrix HG-U133 Plus2.0 array.

**Genes included**	**# of Genes represented**	**Correlation**
all Genes	7,147	0.85

1.5 Fold Change (FC)	3,382	0.90

2.0 FC	2,036	0.92

3.0 FC	1,036	0.933

As intensity values were significantly different due to different formats, we compared the fold change between the universal reference (sample A) and brain (sample B) between the two designs. The correlation between the two platforms was higher when genes that were found to be differentially expressed were considered distinctly, as would be expected where genes showing no differential expression would exhibit a higher variability.

The human GWSA has the added ability to detect alternatively spliced genes due to the extensive design of the probes on the array, so besides measuring gene expression levels, individual splicing events can also be monitored on the array. To investigate the degree of splicing alterations among genes with and without differential gene expression between samples A and B, the set of genes identified as expressed above background were filtered into two groups. One group consisted of genes not differentially expressed between samples A and B (fold changes within the range of -1.2 ≤ x ≤ 1.2), and the other included genes with a greater than 3-fold change (x ≤ -3 or x ≥ 3) between samples. In order to determine the extent of differential splicing that occurred within the two gene categories, a splicing ratio was calculated that consisted of the long form-specific probe set (B) divided by the short form-specific probe set (E) (Additional File 1), called the B/E ratio, and events that had 3-fold change or greater in this ratio between samples A and B were identified. Extensive alternative splicing activity was identified in both groups (Table 3). Those genes that showed high level of differential expression at the gene level (greater than 3 fold change) included 1,844 genes with 13,323 potential splicing events contained within these genes. Of these potential events, 59% (7833) of the events had B/E ratios that were differentially expressed by greater than 3 fold indicating a change in the isoforms for these genes (Table 3). The second category incorporated genes where no overall differential expression change was detected at the gene level (less than 1.2 fold change between the samples A and B), but splicing alterations were identified. 4,434 genes were included within this group and contained 32,917 potential splicing events. 9% (3012) of these events displayed a 3 fold expression change in the B/E ratio suggesting that significant splicing changes were detected in groups of genes regardless of their overall gene expression characteristic demonstrating that transcription and alternative splicing are independent events (Table 3). Within this category of events, approximately 56% were exon(s) skip events and 26% were novel exon events. The other 18% consisted of events detecting intron retentions, novel introns, and alternative splice donor and acceptors. Worth noting, analysis of splice events regardless of the gene expression level revealed that greater than 72% of the splice events that were significantly differentially expressed between samples A and B titrated all four samples correctly further demonstrating the extensive quantification available on the array (data not shown).

**Table 3:** Event analysis of genes grouped by overall gene expression.

**Gene Expression Level** **(median of F and T)**		**Event level** **(B/E ratio)**	
**Fold Change Set**	**Total Genes**	**Total Events**	**Subset of events with ≥3 Fold Change**

x ≥ 3 or ≤ -3	1844	13323	7883

-1.2 ≤ x ≤ 1.2	4434	32917	3012

Genes were classified into two groups: genes that were not differentially expressed between samples A and B (fold changes within the range of -1.2 ≤ x ≤ 1.2), and genes with a greater than 3-fold change (x ≤ -3 or x ≥ 3). In order to determine the differential splicing occurring for each gene, the ratio of the long form probe (B) and the short form probe (E) was calculated, and events were selected where the ratio was differentially expressed between samples A and B by greater than 3-fold.

**Figure 4:**
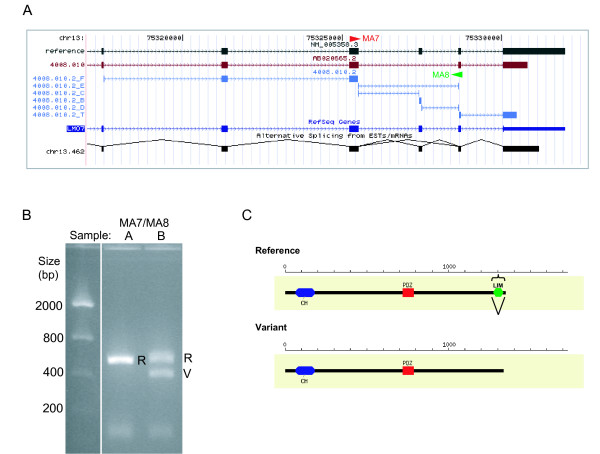
**Lim Domain 7 splicing alteration**. Lim domain 7 (LMO7) was selected from a set of genes that displayed little difference at the gene level between samples A and B, but had event ratios that were significantly higher than 3 fold between these samples, indicating a splicing alteration. **(A) **Using the SpliceArray™ portal () and the associated UCSC browser feature, the event is illustrated with the variant sequence used to identify the event (red), the reference sequence (black), the probe target regions (blue), and the PCR primers (arrowheads) used to validate the event. **(B) **RT-PCR results for the event validation using the primers indicated in A. R = reference and V = variant **(C) **Protein domain analysis was done using CDART [38] and shows the C terminal Lim domain is missing from the splice variant. (Note that Event ID 4008.010 = Entrez Gene ID.ID1 and probe sets are additionally labeled with F, T, B, C, D, or E.)

In order to validate the array analysis and the analytical methods, a total of 40 events were selected from the above mentioned categories. First, events were selected from genes which did not show a significant difference in gene expression between the universal RNA and the human brain RNA samples (median of F and T probe values ≤ 1.2 or ≥ -1.2), but did give a B/E ratio of ≥3, indicating that changes in splicing were occurring. 73% of the events chosen from this list were validated by RT-PCR, and the results showed that the isoform ratio was altered in both samples relative to the expression levels from the human GWSA data. One such event (exon skip) was contained in the LIM domain 7 gene (Figure 4A) which was found to be expressed at similar levels in samples A and B; however, there was an 8 fold change in the B/E ratio for this event. RT-PCR revealed that only the reference or long form was expressed in sample A (universal RNA) while both forms were expressed at approximately equal amounts in sample B (brain, Figure 4B). These results suggest a downregulation of the reference transcript and an upregulation of the variant (exon skip) transcript in sample B compared to sample A. In figure 4B, pooling the reference and variant intensities for each sample would suggest that overall gene level expression is similar in samples A and B which reinforces the idea that examining gene expression alone would mask the shift in the individual transcripts. The monitored event is a deletion of exon 28 (Figure 4A) which causes a predicted frameshift that alters the C-terminal 64 amino acids resulting in the predicted loss of the LIM domain and overall shortening of the protein by 13 amino acids (Figure 4C).

**Figure 5:**
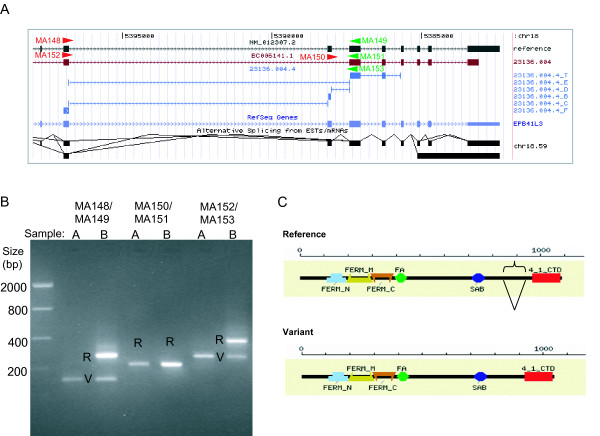
**Erythrocyte membrane protein band 4.1-like 3 (EPB41L3) splicing alteration**. EPB41L3 was selected from a set of genes that displayed a large difference at the gene level between samples A and B, and, in addition, had event ratios that were significantly higher than 3 fold between these samples, indicating a splicing alteration. **(A) **Using the UCSC browser visualization feature of the SpliceArray™ portal (), the event is illustrated with the variant sequence used to identify the event (red), the reference sequence (black), the probe target regions (blue), and the PCR primers (arrowheads) used to validate the event. **(B) **RT-PCR results for the event validation using the primers indicated in A. R = reference and V = variant **(C) **Protein domain analysis was done using CDART [38] and shows a portion of the C terminal region is removed and may affect the function of the protein. (Note that Event ID 23136.004 = Entrez Gene ID.ID1 and probe sets are additionally labeled with F, T, B, C, D, or E.)

Events for validation were also selected from the list of genes which did show significant gene level differences in expression and also showed a change in the B/E ratio of ≥ 3. Overall, all events with conclusive RT-PCR reactions were validated, showing altered isoform ratios in both samples. An example of this class of events is illustrated by the erythrocyte membrane protein band 4.1-like 3 (EPB41L3) gene. At the gene level, EPB41L3 was found to be more highly expressed in brain than in the universal RNA sample, but an exon skip event of exon 17 (Figure 5A) was also found to be altered, evidenced by an 11-fold upregulation of the B/E ratio in brain. RT-PCR analysis clearly indicates that brain RNA (sample B) contains a significantly higher amount of the long form (the reference), while the short form (the exon skip event) is equal or potentially higher in the universal RNA sample (sample A) (Figure 5B). The exon skip event results in an in-frame deletion, leading to the potential loss of 41 amino acids in the C-terminal portion of the protein, between the spectrin-actin domain (SAB), and the 4.1 C-terminal domain (Figure 5C).

## Discussion

Analysis of gene expression has provided researchers with an important resource to identify genes involved in different biological processes, and has been used to generate profiles where the expression level of a predefined set of genes can help to identify and predict a variety of pathological states and prognoses [14,15]. However, many of these studies have ignored the large diversity of transcripts that are generated from each locus by alternative splicing and miss the rich source of diverse transcripts. Researchers have treated each gene as a single entity which is inaccurate considering that potentially over 90% of genes undergo alternative splicing as evidenced by our analysis and recent reports [16,17]. In an attempt to identify different transcripts, studies have been performed where probes have been reordered into new probe sets to define transcripts more accurately [18-20]. However these studies suffer from the same flaw, that the original design of the microarray was not focused on detecting alternative transcripts per se, but to provide an accurate measurement of the overall expression of each gene, not transcript. In contrast, the human Genome-Wide SpliceArray™ (GWSA) is designed to detect alternatively spliced events through the use of exon body and junction probes.

Since Johnson and co-workers [21] published the first human genome-wide microarray that utilized exon-exon junction probes, several groups have simultaneously produced microarrays to monitor splice events using similar probe designs [11,22-24]. The GWSA microarray is the most comprehensive design commercially available with over 6 million probes. The array design consists of a maximum of 6 probe types targeted to the exon body, exon-exon junctions, and exon-intron/intron-exon junctions allowing more complete detection of transcripts, in contrast to those commercially available arrays that incorporate only exon body probes [25] or only junction probes [24]. The SpliceArray™ probe design simultaneously monitors both isoforms for each splice event and is able to detect alternative exon, intron retention, alternative splice acceptor (ASA), and alternative splice donor (ASD) events. Additionally, the design has the ability to monitor putative novel skipped exons and unidentified ASA and ASD events. Although the design is theoretically capable of detecting all types of events, it does have the limitation where very short sequence differences (less than 8 bases) are not captured and these events have been filtered out of the collection. In addition, the microarray platform itself has limited space and even at the maximum number of features, only 6.54 million probes can be included which limits the number of probes per probe set that can be designed for any event whether evidenced or predicted. Furthermore, microarray platforms possess limitations on the sensitivity to detect very small changes in transcript expression. A consequence of manufacturing the GWSA content on the Affymetrix platform is that the cost for updating the array probe content is significant as it would require re-manufacturing a custom array and therefore recent discoveries of novel splice events cannot feasibly be added to the design. However, it should be pointed out that the GWSA monitors ~140,000 predicted exon skip events so although there was no evidence of these exon skip events at the time the GWSA content was generated, it indicates that the array has the potential to monitor newly discovered exon skip events.

Concurrent with the generation of microarray designs able to detect alternatively spliced transcripts, many analytical methods have been developed to determine the extent of alternative splicing and identify the expression level for each transcript within a gene. We used a simple approach to identify cases where the ratio of the inclusive and exclusive isoforms changed dramatically, by calculating the ratio of probes that were specific for the inclusive or long form (probe B) versus probes specific for the exclusive or short form (probe E). This approach was shown to provide very powerful results when assessed against PCR validation of the events. It is a simple and applicable approach that provides strong filtering methods for real positives, unlike other methodologies which require an extensive programming and array fitting [22].

Performance of the human GWSA product was evaluated in this study and the arrays were shown to be highly reproducible, suggesting that the reproducibility is more a function of the platform and labeling procedures than of the probe design. Analysis of the array data at the gene level proved highly concordant with data produced in the MAQC project on the HG-U133 Plus 2.0 array. Even though the platforms were slightly different (the HG-U133 Plus 2.0 array contains 11 micron features while the GWSA contains 5 micron features), the genes showed similar levels of expression, and importantly, comparable quantified changes between the universal and brain RNA samples. High concordance was observed between these similar platforms even though the probe sets were designed for different applications. Additionally, the GWSA and other SpliceArray™ products also provide splice event information available from the same experiment, exemplified by several recent reports [3,4,26-29].

Validation of specific splice events from the GWSA hybridizations was performed based on the analysis of the ratio of the long isoform to the short isoform. This type of assessment is an important aspect to determining the extent of alternative splicing as in many cases the ratio of isoforms will determine the biological response [30-33]. By assessing the statistical power of the ratio, we were able to validate a high rate of events; overall, 81% of statistically selected events were validated by RT-PCR. The high rate of validation was found regardless of the overall transcriptional changes identified. Interestingly, almost 60% of the events identified to have gene level, transcriptional alterations of 3 fold or more were found to have an event fold change of 3 fold or more. This indicates that not only is there a change in overall gene expression, but the transcripts produced are different as generated by alternative splicing. Surprisingly, genes that showed no change in their expression levels represented a rich source of alternatively spliced transcripts. Almost 10% of the events among these genes contained evidence of splicing alterations by changes in the ratio of the different isoforms. This is an important finding as it brings to point that genes which have been ignored because of equivocal expression between samples actually have important changes that occur in producing different isoforms. This confirms earlier evidence [34] that illustrates transcriptional activity is independent of splicing, even though the two processes function simultaneously and emphasizes the need to measure both overall gene expression and alternatively spliced transcripts for greater understanding of biological processes. The above findings should encourage researchers to look more closely at the genes which show no variation in overall gene expression for important clues into the mechanisms in pathophysiology and biology.

Alternative splicing affects not only the structure of the mRNA but ultimately the structure of the protein produced. Many exons encode protein domains that can be removed by an exon skip event [35]. Such an event can produce proteins that lack a functional domain, dominant negative proteins, constitutively active isoforms, soluble homologues, or can lead to the regulation of the protein's overall activity by altering its global expression. The two examples, erythrocyte membrane protein band 4.1-like 3 (EPB41L3), and Lim domain 7 (LMO7) depicted here show how changes at the transcript level can be observed with or without gene expression changes, respectively. Multiple alternative splice variants have been previously described for LMO7 [36] and the variant detected here results in deletion of the Lim domain, a protein-protein interaction domain found in many key regulators of development. EPB41L3 is potentially a critical growth regulator in meningioma pathogenesis [37] and is normally expressed at high levels in brain, with lower levels in kidney, intestine, and testis. The function of the EPB41L3 variant is unknown, yet contains some well described protein domains, including Ferm domains found in many cytoskeletal proteins, the N domain found in ubiquitin-like structural domain, and the C domain found in tyrosine phosphatases [38]. Different isoforms certainly will affect the function of these different domains and potentially the protein, and may play a role in pathological states.

## Conclusion

The identification of expressed transcripts is at the heart of expression analysis and these examples, as well as recently identified novel spliced isoforms demonstrating diverse activity [4,6] illustrate the importance of correct determination of the expression of each transcript. Array platforms specifically designed to detect the differences in transcripts along with other novel technologies [39] will allow researchers to more fully explore the transcriptome under different physiological and pathological conditions. In short, we have provided a unique platform to detect isoform diversity in the human transcriptome by utilizing algorithmically designed novel exon body and splice junction probes to detect every possible isoform event. This approach on a genome-wide scale has been demonstrated to be highly reproducible and quantitative.

## Methods

### Materials

Total RNA was purchased from Stratagene (Universal Human Reference RNA, # 740000; sample A) (LaJolla, CA, USA) and Ambion (Human Brain Total RNA, # AM7962; sample B) (Austin, TX, USA). Total RNA quality and concentration were verified using the Agilent 2100 bioanalyzer (Agilent, Santa Clara, CA, USA). Four titration samples (A, B, C = 75%A+25%B, and D = 25%A+75%B) were generated as previously described [13] and each sample was analyzed in triplicate.

The content for the human Genome-Wide SpliceArray™ (GWSA) was designed using Build 35 of the NCBI Human (genome) reference sequence produced by the International Human Genome Sequencing Consortium and sequence data from the NCBI GenBank sequence database. Additional information was used from the NCBI Entrez Gene database in the representation of the genes. For each gene, a RefSeq was selected as the reference form, the selection considered which RefSeq (when more than one was available) covered the largest genomic area to maximize sequence inclusion (based on mapping against a genomic range overlapping the selected representative). Sequences used in the analysis were aligned to the human genome and data from this alignment was used to create distinct exon structures. Significant differences from the reference sequence were detected and probes were designed to monitor each difference or splice event as previously described [11]. All splice events included on the GWSA were subject to manual review to remove those events that appeared to be indicated by sequence or alignment artifact. Additionally, splice events were removed with the following characteristics: those that resulted in very small changes (of a few nucleotides) to the transcript; where the required probes would not conform to Affymetrix manufacturing standards; and that would not be detected by the SpliceArray™ probe configuration. The human GWSA custom microarrays were manufactured by Affymetrix (Santa Clara, CA, USA) as a 49 format, 5 micron array containing over 6 million probes which monitor approximately 229,000 exons and 181,000 junctions. GC probe sets for background correction and positive and negative control probes were included on the array. The probes on the array are designed in the sense orientation and for use with the NuGEN WT-Ovation™ RNA Amplification Systems and FL-Ovation™ Biotin Module Products. For additional information on SpliceArray products and probe design, refer to .

### MicroArray processing: Transcript amplification and labeling

Amplified cDNA was prepared using the WT-Ovation™ Pico RNA Amplification System (NuGen product #3300; Santa Clara, CA, USA) starting with 50 ng of each total RNA sample, as described by the manufacturer. Briefly, the RNA is synthesized into double-stranded cDNA using a SPIA™ DNA/RNA primer. The DNA portion of the primer binds poly (A) sequence or randomly across the transcript, and the RNA portion of the primer is incorporated into an unique DNA/RNA heteroduplex at one end of the cDNA. RNase H degrades the RNA portion of the DNA/RNA heteroduplex allowing for additional SPIA™ primer to bind and initiate replication by DNA polymerase. The process of SPIA™ DNA/RNA primer binding, DNA replication, strand displacement and RNA cleavage is repeated resulting in isothermal linear amplification of the cDNA. On average, the cDNA yield from 50 ng of high quality total RNA ranges from 6 to 10 micrograms. Fragmentation and biotin-labeling of the amplified cDNA was performed using the FL-Ovation™ cDNA Biotin Module V2 (NuGen product #4200) as per manufacturer's instructions.

### Array hybridization, scanning, and data extraction

Prepared target for each sample was hybridized to the Affymetrix-formatted SpliceArray™ product using standard hybridization methods recommended by the manufacturer for the 49-format (Affymetrix - Expression analysis technical manual). The arrays were stained and washed using the Affymetrix FS450-0001 fluidics protocol prior to scanning with the Affymetrix GeneChip^® ^Scanner 3000 7G. DAT and CEL images were visually inspected for anomalies and accurate grid placement. All data are available through the Gene Expression Omnibus (GEO) database as series accession number GSE17258.

### Data analysis

The analysis described here was generated using Partek^® ^software (). For the comparative analysis, data for the HG-U133 Plus 2.0 GeneChip was taken from the MAQC data set publicly available at . All array data was pre-processed which included probe level RMA background correction, quantile normalization across all arrays, and Log2 transformation followed by median polish to summarize probes to obtain the overall score for each probe set. The data were filtered based on the expression values of each probe set within the triplicate set for each sample; if the expression value of a probe set was below 3.5 (log 2 value) the probe set was removed from the analysis. In order to identify probe sets that were significantly different between samples A and B, a one-way ANOVA statistical test was performed on normalized and filtered probe set level intensity values between each group to generate p-value and fold change data.

To identify genes that were significantly different between samples A and B, a list of non-redundant evidenced probe sets were imported into Partek^® ^software. Non-logged median values for F and T evidenced probe sets with the same Entrez Gene ID were calculated to generate an overall gene score for each gene. After removing genes which were expressed at a low level, a one-way ANOVA statistical test was then performed on the gene level intensity values between sample groups A and B to generate p-values and fold changes for each gene.

In order to estimate gene level correlation between the human GWSA and the HG-U133 Plus 2.0 array data, both data sets were imported separately and subjected to the same pre-processing method. For the GWSA data, a gene score for each sample was generated as described above. After separately filtering the low-expressing genes in both data sets, a total of 7,147 common genes were identified. The log value of the ratio of sample A/sample B of gene values was calculated for each gene and platform, and a linear correlation coefficient was calculated between the GWSA and HG-U133 Plus 2.0.

Event level analysis performed using Partek^® ^software was based on the calculated ratios between the B probe (specific to the inclusive isoform or long form) divided by the E probe (specific to the exclusive or short isoform). This ratio can be considered a splice score, but has no units. Only when comparing one sample to another can an interpretation be made about the differential expression of the splice variants. A ratio between the non-logged B and E probe set intensities for each sample was computed based on normalized and filtered probe set data. Since the reference and the variant transcripts are monitored by separate probe sets (B and E) and it is the ratio being calculated between the two probe sets, there is no need to normalize for overall gene expression.



A one-way ANOVA statistical test was subsequently performed on the log 2 based ratios between each of the sample groups to generate p-values and fold change of the B/E ratio between samples A and B. Similar methods were used to calculate C/E and D/E ratio, which provided similar data.

Using the human GWSA gene level intensity data, two lists were generated, those genes that displayed no differential expression change (-1.2 ≤ X ≤ 1.2) between samples A and B and a second group showing significant gene expression change, greater than 3 fold change. ANOVA analysis identified events within these two separate groups having significant B/E ratios between samples A and B. Top events for RT-PCR validation were selected by choosing events with the highest fold changes, p-values < 0.001, and that titrated all 4 samples correctly.

### RT-PCR Processing

First strand cDNAs were prepared using a random priming protocol starting with 5 μg of the individual RNA samples using the High Capacity cDNA Archive Kit (Applied Biosystems; Foster City, CA, USA). cDNA was prepared from both the human normal brain and universal human reference RNA samples and the quality and consistency were assessed by determining the levels of ribosomal protein P0 by RT-PCR. Additionally, samples were normalized by the level of ribosomal P0 for validation of each splice event.

Lasergene/Primer Select (DNAstar) software was used for primer design. Primer sequences will be provided upon request. RT-PCR amplification was performed using a cDNA dilution with the equivalent of 17 ng of total RNA for each reaction. Promega 2× PCR Master Mix (Madison, WI, USA) was used for each 50 ul reaction with forward and reverse primer at a final concentration of 0.3 uM. A "touchdown" cycling program was used to amplify products (annealing temp 65°C-60°C, 5 cycles, and 60°C, 35 cycles). 12 μl of PCR products were analyzed using either 2% Agarose or 3% Metaphor gels.

## Competing interests

All authors are employees of ExonHit Therapeutics, a drug discovery and diagnostic development company. The manuscript describes a major technology used for internal and extramural programs for the company. A conflict of interest exists due to the financial interest that the authors have in ExonHit Therapeutics, Inc.

## Authors' contributions

RE, MB, SJ, DP, CS and FR designed the array content and carried out the bioinformatics analysis. DW, LL and HJ carried out the experiments and collected the data. WZ and RE conceived of new analytical methods for the analysis, and WZ, PF and RE performed data analysis. RE conceived of the study and MC, WZ, LB, HJ, PG and RE wrote the paper. All authors read and approved the final manuscript.

## Supplementary Material

Additional file 1 **Probe configuration for the human Genome-Wide SpliceArray™ 1 figure with corresponding legend.** Click here for file

Additional file 2 **Sample analysis on the human GWSA**. (A) Frequency Distribution (B) Principal Component Analysis. Click here for file
