# Supplementary material for: High resolution analysis of the human transcriptome: detection of extensive alternative splicing independent of transcriptional activity
Source: BMC Genet. 2009 Oct 5;10:63. doi: 10.1186/1471-2156-10-63 (PMC2768739; doi:10.1186/1471-2156-10-63)
Supplement: Additional file 1 — Probe configuration for the human Genome-Wide SpliceArray™ 1 figure with corresponding legend. [file 1471-2156-10-63-S1.DOC]

**Additional File 1:**

**
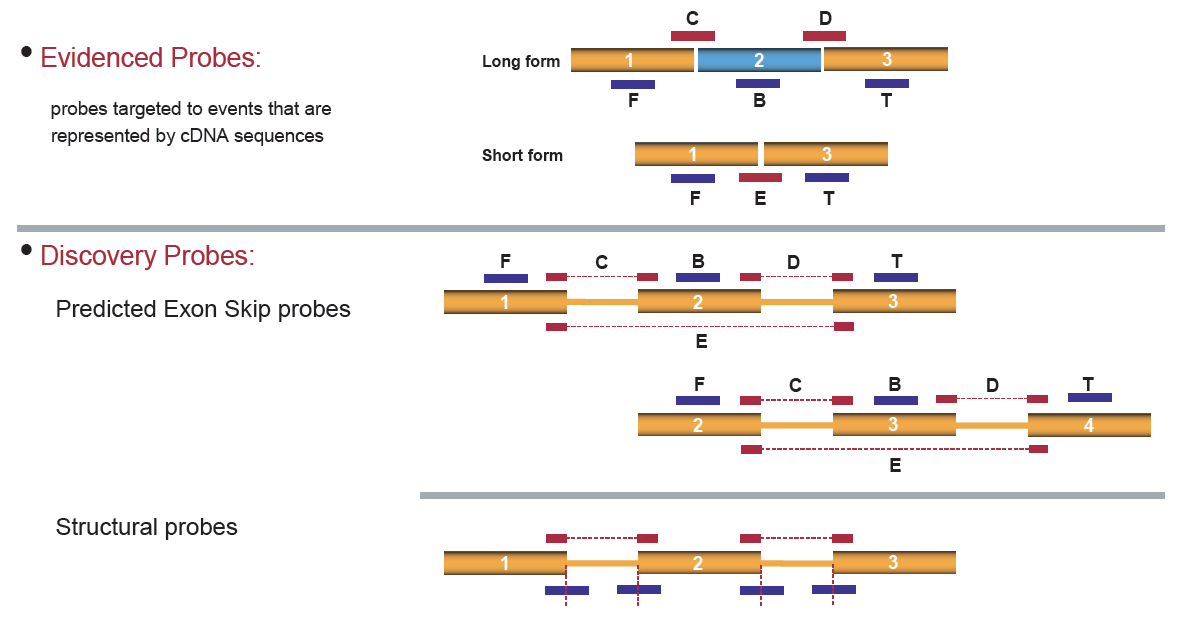
**

**Figure:** **Probe configuration for the human Genome-Wide SpliceArray^TM^.** Potential splice events were identified after investigation of expressed sequence from the public databases. These “evidenced events” were selected and probes were designed to detect both the short, exclusive form and the long, inclusive form. F and T probe sets are common to both forms while B, C, and D probe sets are specific to the long form and the E probe set is specific only for the short form. There are two types of discovery probes which include predicted exon skips and structural probes. If an exon was not detected to be skipped from the sequence analysis, then the exon was predicted to be skipped and probes were designed to detect each prediction. Structural probes are designed against the exon-intron structure of the gene, such that each intron contains three probe sets; an exon-exon junction, an exon-intron and intron-exon probe set to monitor exon extension events.
