# Supplementary material for: High resolution analysis of the human transcriptome: detection of extensive alternative splicing independent of transcriptional activity
Source: BMC Genet. 2009 Oct 5;10:63. doi: 10.1186/1471-2156-10-63 (PMC2768739; doi:10.1186/1471-2156-10-63)
Supplement: Additional file 2 — Sample analysis on the human GWSA. (A) Frequency Distribution (B) Principal Component Analysis. [file 1471-2156-10-63-S2.DOC]

**Additional file 2**

**
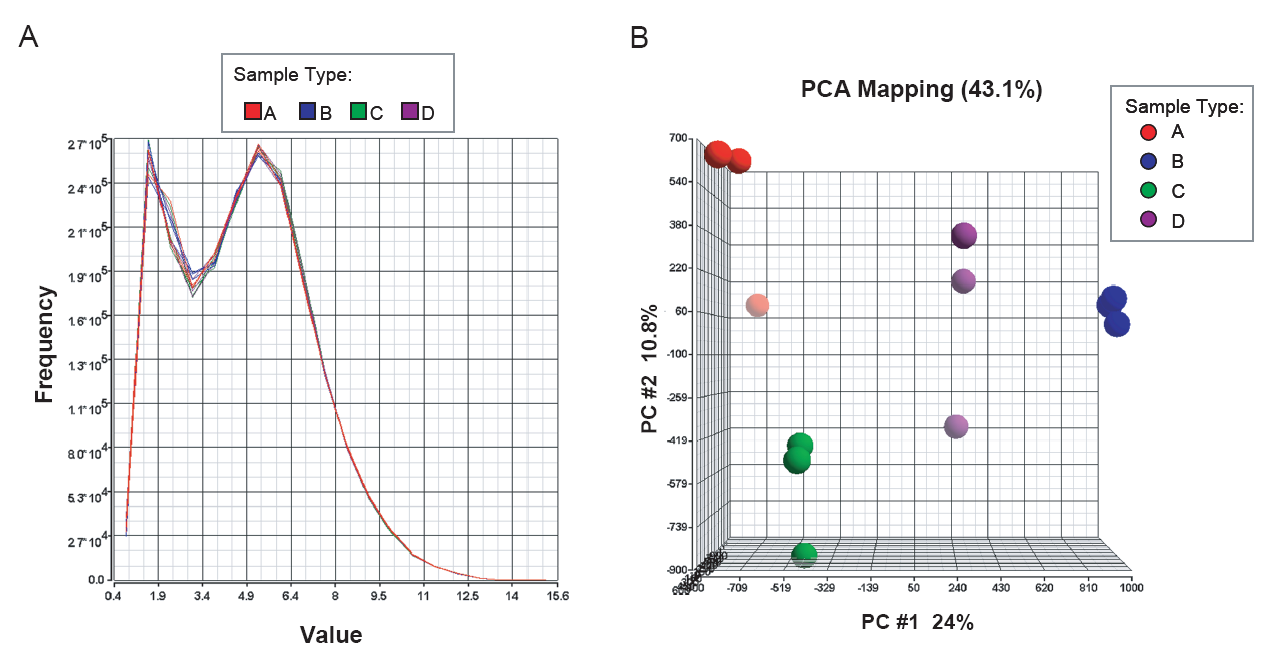
**

**Figure:** **Sample analysis on the human GWSA.** The four samples hybridized include A= universal reference, B= total brain, C = 75%A+25%B, and D= 25%A+75%B. Samples were run in triplicate. **(A)** Frequency distribution is shown after normalization of the entire data set. **(B)** Principal Component Analysis (PCA) was performed on the normalized distribution and displays the correct orientation of the samples (sample A on the left, followed by samples C, D, and B moving to the right), as well as good reproducibility among the replicates on the array.
